# 150 Workplace physical activity practices for small-and medium-sized enterprises: identification of features that work in real life

**DOI:** 10.1093/eurpub/ckae114.195

**Published:** 2024-09-26

**Authors:** Frank Vandaele, Anna Puig-Ribera, Ilkka Väänänen, Estela Farías-Torbidoni, Víctor Dorado, Sergi Matas, Anna Codina-Nadal, Eva Aumatell, Ine De Clerck, Sebastià Mas-Alòs

**Affiliations:** Artevelde University Of Applied Sciences, Gent, België; University of Vic-Central University of Catalonia, Spain; LAB University of Applied Sciences, Lahti Campus, Finland; National Institute of Physical Education of Catalonia, Spain; University of Lleida (UdL), GISEAFE Research Group, Spain; National Institute of Physical Education of Catalonia, Spain; University of Lleida (UdL), GISEAFE Research Group, Spain; National Institute of Physical Education of Catalonia, Spain; University of Lleida, Human Movement Research Group, Spain; University of Vic-Central University of Catalonia, Spain; Open University of Catalonia, eHealth Centre, Barcelona, Spain; Artevelde University Of Applied Sciences, Gent, België; National Institute of Physical Education of Catalonia, Spain; University of Lleida, Human Movement Research Group, Spain

## Abstract

**Background and Objectives:**

In the current landscape of workplace well-being policies, the promotion of adequate physical activity (PA) has gained prominence. Extensive peer-reviewed literature outlines essential components for effective PA initiatives. Building upon Väänänen et al.’s (2022) scoping review of grey literature, this study aims to assess which of the identified initiatives are most feasible for inclusion in Small and Medium-sized Enterprises (SMEs) in the European Region of the World Health Organization.

**Methods:**

A questionnaire to evaluate the feasibility was administrated to 82 experts from 26 countries, including the Worksite HEPA promotion working group and experts contacted by the WHO Regional Office for Europe (HEPA focal points). These experts rated the initiatives on positive attributes for practice transfer (effectiveness, practicality, adaptability, generalizability, and cost-benefit) using a 5-point Likert scale. Ratings were categorized by company size, and experts provided recommendations for their countries or regions on a scale of 1-10. Subsequently, an online Delphi panel of experts gathered qualitative feedback on the initiatives with the highest scores, facilitating consensus for their inclusion in a European PA Toolkit tailored for SMEs.

**Results:**

Through three waves, 28 experts assessed a total of 390 initiatives. Forty-three initiatives were considered most feasible for SMEs because they reached a minimum score of 20/25 on positive attributes for practice transfer plus any attributed scored below 4. These initiatives were appraised feasible for SMEs, obtaining a recommendation score of at least 7. After two rounds of Delphi panels involving six experts, no consensus was reached on which of the 43 initiatives were most feasible for SMEs.

**Conclusions:**

The 43 initiatives that were considered feasible through the questionnaire-based assessment, covered all Workplace PA initiative categories identified by Väänänen et al. 2022 (Active work and living; Exercise and fitness programs; Management and leadership; Communication and dissemination; and Facilities). The experts found the underlying concept of these initiatives inspiring for European SMEs. However, they are not transferable to every context. Further research is needed to determine the conditions that must be met to make these concepts feasible across various contexts.

**Funding Source:**

World Health Organization Regional Office for Europe

